# Supplementary material for: Insights on autophagosome–lysosome tethering from structural and biochemical characterization of human autophagy factor EPG5
Source: Commun Biol. 2021 Mar 5;4:291. doi: 10.1038/s42003-021-01830-x (PMC7935953; doi:10.1038/s42003-021-01830-x)
Supplement: Supplementary file 2 — Supplementary Information [file 42003_2021_1830_MOESM2_ESM.pdf]

Insights on autophagosome-lysosome tethering from structural and biochemical characterization of human autophagy factor EPG5

Sung-Eun Nam[1+], Yiu Wing Sunny Cheung[1+], Thanh Ngoc Nguyen[2], Michael Gong[1], Samuel Chan[1], Michael Lazarou[2], Calvin K. Yip[1*]

Supplementary Information

Supplementary Figure 1. Purification of the hEPG5 constructs.

Supplementary Figure 2. *In vitro* pulldown assays of hEPG5 mutants and the LC3/GABARAP subfamily.

Supplementary Figure 3. Comparison of the LIR2-GABARAPL1 crystal structure with previously published apo- and LIR bound-GABARAPL1 crystal structures.

Supplementary Figure 4. Gold-standard Fourier shell correlation curve.

Supplementary Figure 5. Uncropped blots for Figure 2 and 6

Supplementary Figure 6. Uncropped blots for Supplementary Figure 2

Supplementary Table 1. Thermodynamic parameters of ITC experiments of human ATG8 proteins with hEPG5-LIR1 and LIR2 peptides.

Supplementary Table 2. Negative stain EM datasets and 2D analysis details.

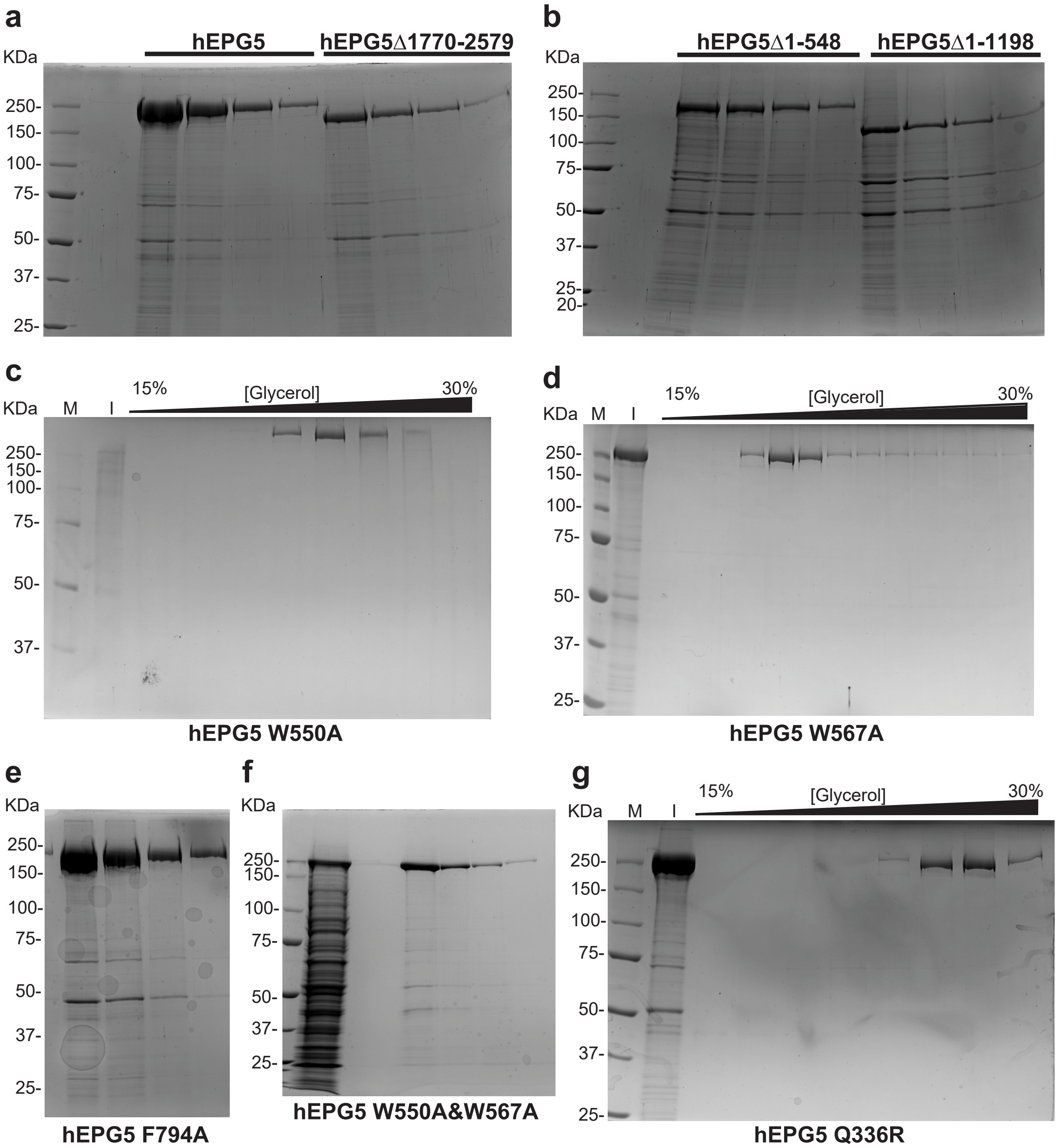

Supplementary Figure 1. Purification of the hEPG5 constructs. a-g 6%-15% SDS-PAGE of His-FLAG-hEPG5 mutants isolated by anti-FLAG affinity chromatography and glycerol gradient ultracentrifugation fractions, stained with Coomassie Blue. M and I represent the protein marker and input, respectively. **(a)** Elution fractions of wild type hEPG5 and C-terminal truncation mutant hEPG5$^{\Delta 1770\text{-}2579}$. **(b)** Elution fractions of hEPG5 N-terminal truncation mutant hEPG5$^{\Delta 1\text{-}548}$ and hEPG5$^{\Delta 1\text{-}1198}$. **(c)** hEPG5 mutant with LIR1 aromatic residue Trp550 mutated to alanine. **(d)** hEPG5 mutant with LIR2 aromatic residue Trp567

mutated to alanine. **(e)** Elution fractions of hEPG5 mutant with LIR3 aromatic residue Phe794 mutated to alanine. **(f)** Elution fractions of hEPG5 double mutant with both LIR1 aromatic residue Trp550 and LIR2 aromatic residue Trp567 mutated to alanine. **(g)** hEPG5 Vici syndrome mutant with residue Gln336 mutated to arginine.

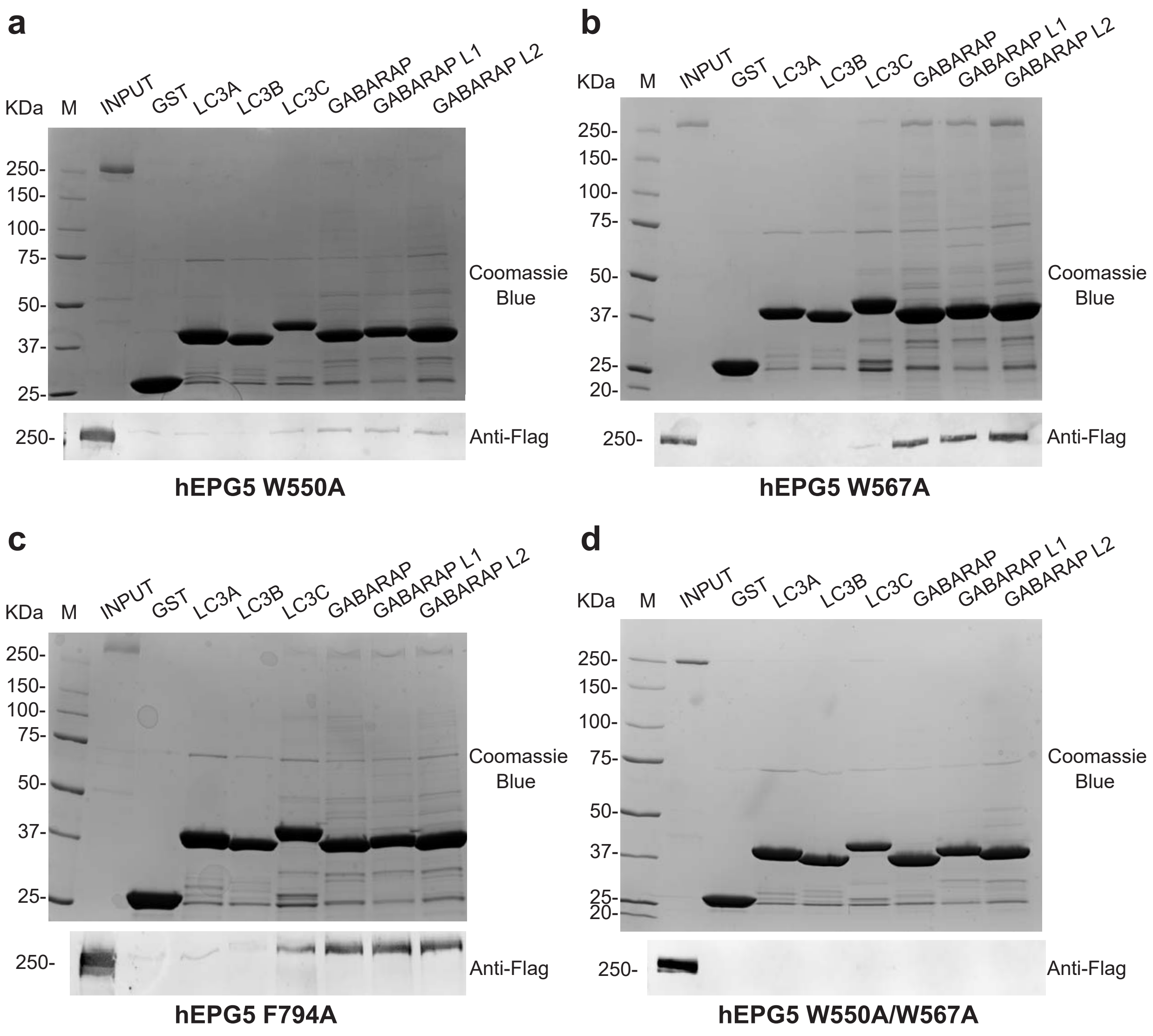

Supplementary Figure 2. *In vitro* pulldown assays of hEPG5 mutants and the LC3/GABARAP subfamily. a-d *In vitro* pulldown assays of hEPG5 mutants with GST (control) and GST-tagged LC3/GABARAP subfamily proteins used as baits. Representative 6%-15% SDS-PAGE stained with Coomassie Blue (top panel) shows the hEPG5 mutants and GST-tagged LC3/GABARAP input, and representative Western blot (bottom panel) was probed by anti-FLAG antibodies. M represents the protein marker. Experiments were performed in triplicates. **(a)** hEPG5^{W550A} mutant was used as prey. **(b)** hEPG5^{W567A} mutant was used as prey. **(c)** hEPG5^{F794A} mutant was used as prey. **(d)** hEPG5$^{W550A/W567A}$ mutant was used as prey.

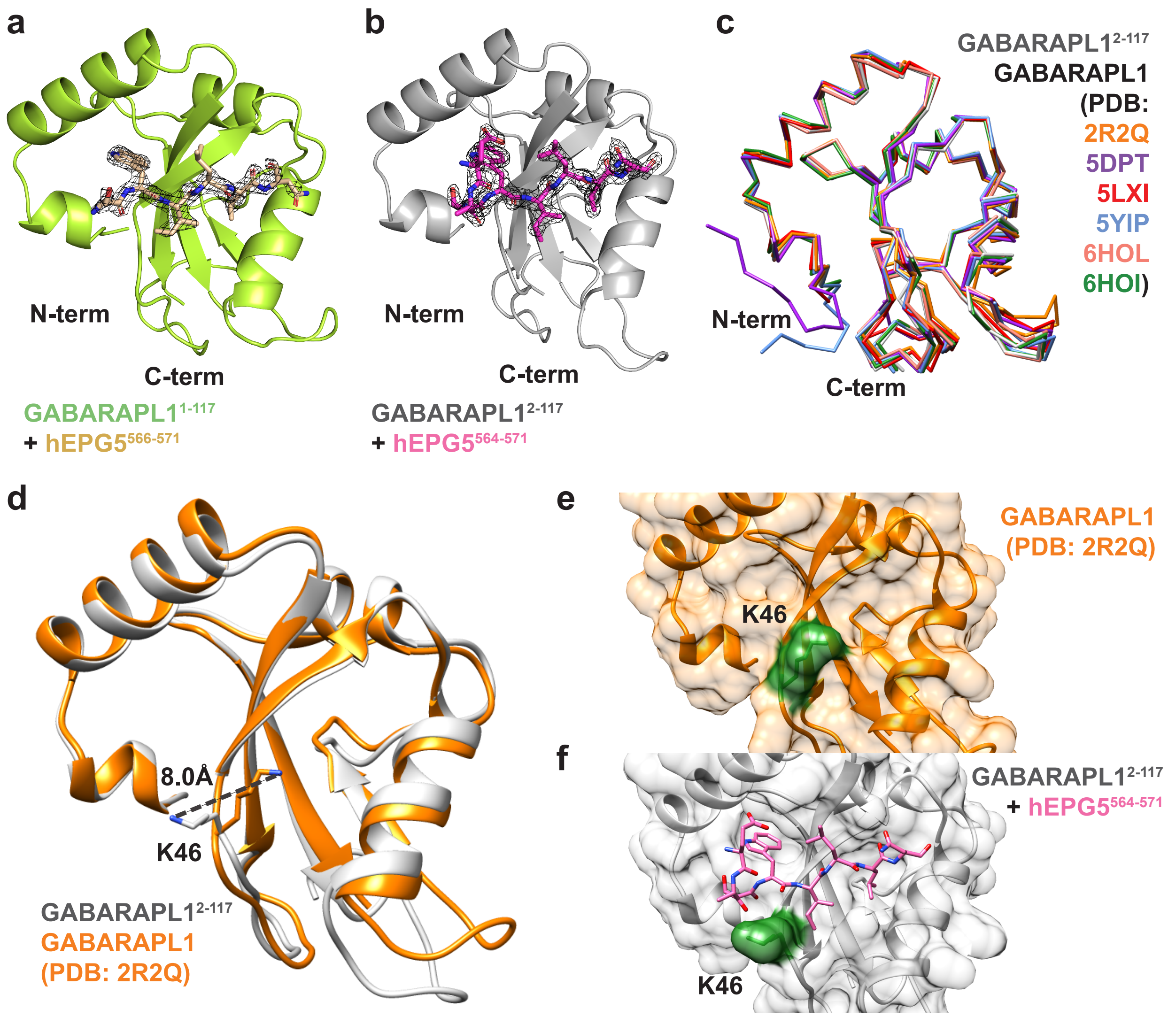

Supplementary Figure 3. Comparison of the LIR2-GABARAPL1 crystal structure with previously published apo- and LIR bound-GABARAPL1 crystal structures.
a, b Refined 2Fo-Fc map of hEPG5-LIR2 in the two LIR2-GABARAPL1 complexes present in the crystal asymmetric unit, contour at $\sigma = 1.5$. **(a)** GABARAPL1$^{1\text{-}117}$ (molecule A) in complex with LIR2 (hEPG5$^{566\text{-}571}$) (molecule C) are represented in light green cartoon and gold sticks, respectively. **(b)** GABARAPL1$^{2\text{-}117}$ (molecule B) in complex with LIR2 (hEPG5$^{564\text{-}571}$) (molecule D) are represented in grey cartoon and pink sticks, respectively. **c** Superimposition of GABARAPL1 proteins. GABARAPL1 (grey; molecule B in this study) is superimposed with the apo-GABARAPL1 (orange; PDB:2R2Q) and previously published LIR bound-GABARAPL1, including PLEKHM1 LIR-GABARAPL1 (purple; PDB:5DPT), ATG4B LIR-GABARAPL1 (red; PDB:5LXI), AnkG LIR-GABARAPL1 (light blue; PDB:5YIP), ATG14 LIR-GABARAPL1 (pink; PDB:6HOL) and Beclin1 LIR-GABARAPL1 (green; PDB:6HOI). **d** GABARAPL1 residue Lys46 sidechain undergoes a conformational rearrangement upon LIR2 binding. GABARAPL1 (grey) is superimposed with apo-

GABARAPL1 (orange; PDB:2R2Q). Residue Lys46 sidechain shifts outwards by 8.0 Å upon LIR2 binding. **e** Close-up view apo-GABARAPL1. Ribbon and transparent surface representation of apo-GABARAPL1 (orange; PDB:2R2Q), with residue Lys46 highlighted in green. **f** Close-up view LIR2 bound-GABARAPL1. Ribbon and transparent surface representation of GABARAPL1 (grey) with LIR2 (pink) bound. Residue Lys46 is highlighted in green. The conformational rearrangement of Lys46 creates space to accommodate LIR2 residue I568 binding.

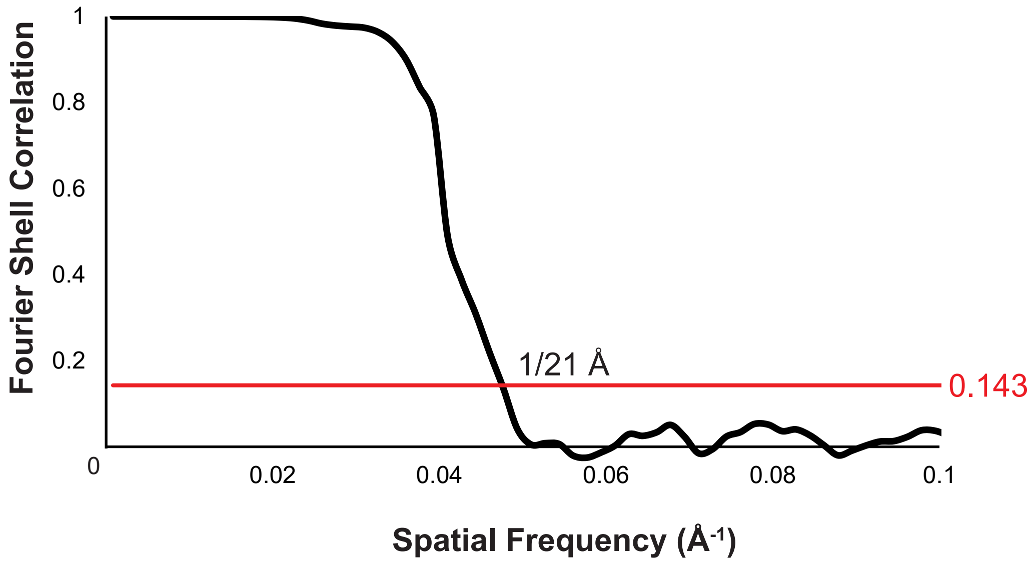

Supplementary Figure 4. Gold-standard Fourier shell correlation curve. The estimated resolution of the refined hEPG5 3D reconstruction is 21 Å using the 0.143 criterion.

Fig. 2a

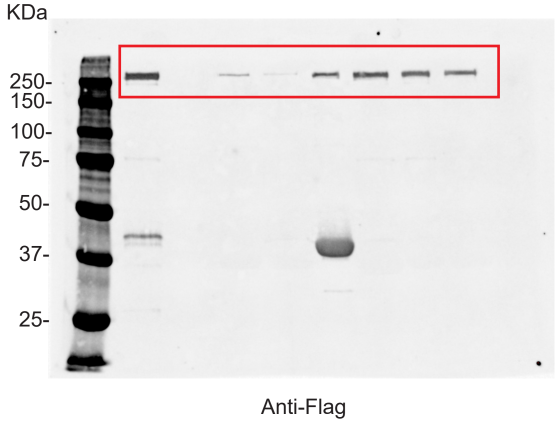

Fig. 2c

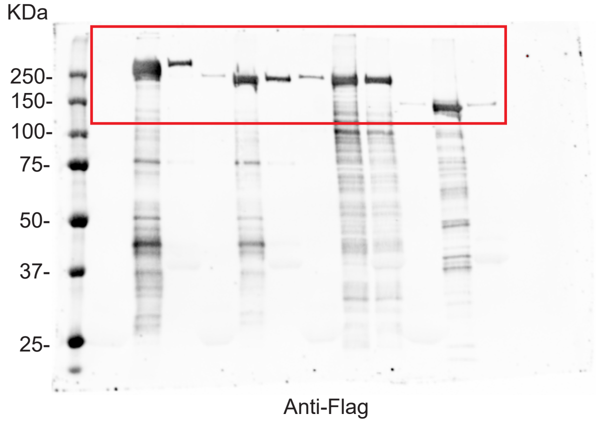

Fig. 2e

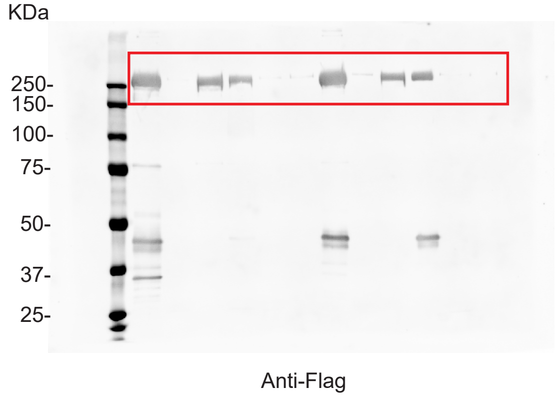

Fig. 6d

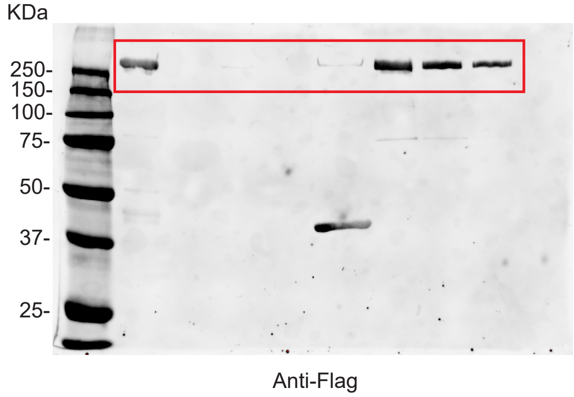

Supplementary Figure 5 | Uncropped blots for Figure 2 and 6.

Supplementary Fig. 2a

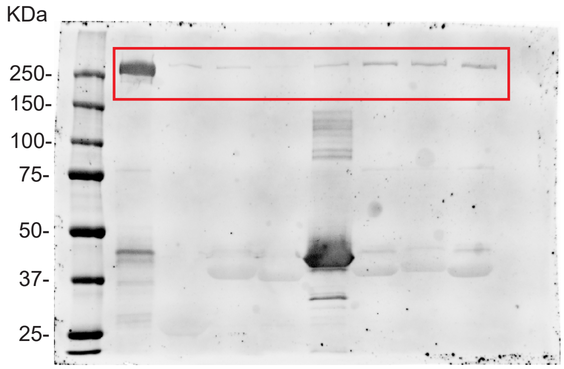

Anti-Flag

Supplementary Fig. 2b

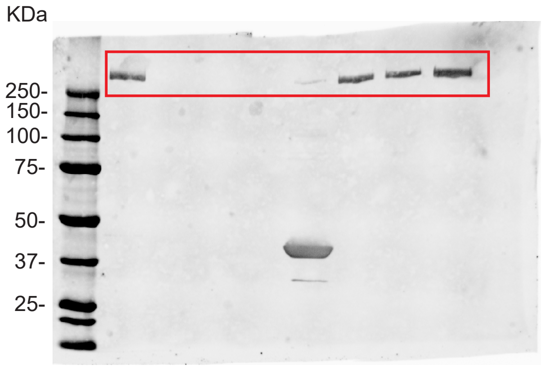

Anti-Flag

Supplementary Fig. 2c

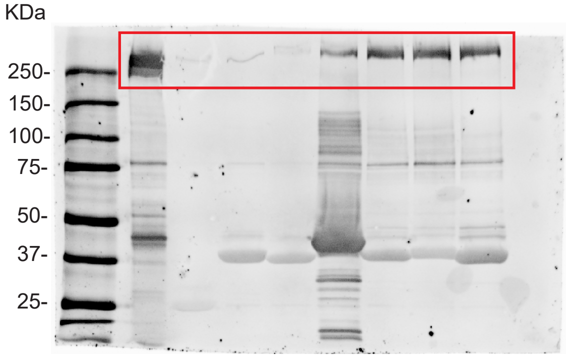

Anti-Flag

Supplementary Fig. 2d

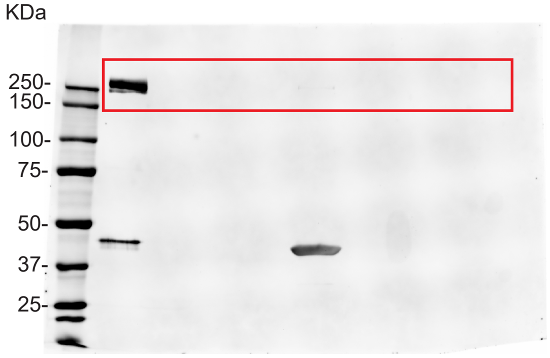

Anti-Flag

Supplementary Figure 6 | Uncropped blots for Supplementary Figure 2.

Supplementary Table 1. Thermodynamic parameters of Isothermal Titration Calorimetry (ITC) experiments of human ATG8 proteins with hEPG5-LIR1 and LIR2 peptides.

	ΔH (kcal mol^{-1})	ΔS (cal mol^{-1} K^{-1})	ΔG (kcal mol^{-1})	K_A ($x10^6$ M^{-1})	K_D (µM)	*N*
**hEPG5-LIR1 (GSGTWTLVDEG)**						
**LC3A**	-4.7 ± 0.7	5.55	-6.40	0.05 ± 0.01	20.38	1.06 ± 0.16
**LC3B**	No Binding Determinable					
**LC3C**	No Binding Determinable					
**GABARAP**	-6.8 ± 0.4	0.65	-7.00	0.14 ± 0.03	7.47	0.96 ± 0.09
**GABARAPL1**	-6.1 ± 0.4	2.78	-6.92	0.12 ± 0.01	8.54	0.85 ± 0.22
**GABARAPL2**	-4.2 ± 0.4	8.68	-6.73	0.09 ± 0.02	11.79	0.94 ± 0.02
**hEPG5-LIR2 (DEDPETSWILLN)**						
**LC3A**	-3.6 ± 0.1	14.4	-7.87	0.58 ± 0.10	1.75	0.93 ± 0.03
**LC3B**	-3.5 ± 0.3	13.1	-7.36	0.25 ± 0.02	4.07	0.94 ± 0.05
**LC3C**	No Binding Determinable					
**GABARAP**	-5.6 ± 0.2	12.3	-9.30	6.79 ± 2.42	0.16	0.93 ± 0.04
**GABARAPL1**	-5.2 ± 0.4	14.8	-9.64	11.77 ± 0.72	0.09	0.90 ± 0.01
**GABARAPL2**	-2.0 ± 0.2	21.6	-6.44	1.57 ± 0.47	0.68	0.84 ± 0.04

Supplementary Table 2. Negative stain EM datasets and 2D analysis details.

Protein/Protein complex	Total number of particles	Number of 2D classes specified	Number of particles in representative image
**hEPG5 (Fig. 1d)**	10,866	50	1,581
**hEPG5$^{\Delta 2079\text{-}2579}$ (Fig. 1e)**	127	4	48
**hEPG5-MBP (Fig. 1f)**	3,167	25	62
**hEPG5-GABARAP (Fig. 2d)**	3,253	18	279
**hEPG5^{Q336R} (Fig. 6a)**	6,574	15	778

Supplementary References

1. Rogov, V. V. *et al*. Structural and functional analysis of the GABARAP interaction motif (GIM). *EMBO Rep*. **18**, 1382–1396 (2017).
2. Skytte Rasmussen M. *et al*. ATG4B contains a C-terminal LIR motif important for binding and efficient cleavage of mammalian orthologs of yeast Atg8. *Autophagy*. **13**, 834–853 (2017).
3. Li, J. *et al*. Potent and specific Atg8-targeting autophagy inhibitory peptides from giant ankyrins. *Nat. Chem. Biol*. **14**, 778–787 (2018).
4. Birgisdottir, Å. B. *et al*. Members of the autophagy class III phosphatidylinositol 3-kinase complex I interact with GABARAP and GABARAPL1 via LIR motifs. *Autophagy*. **15**, 1333–1355 (2019).
