# Supplementary material for: Insights on autophagosome–lysosome tethering from structural and biochemical characterization of human autophagy factor EPG5
Source: Commun Biol. 2021 Mar 5;4:291. doi: 10.1038/s42003-021-01830-x (PMC7935953; doi:10.1038/s42003-021-01830-x)
Supplement: Supplementary file 3 — Description of Additional Supplementary Files [file 42003_2021_1830_MOESM3_ESM.pdf]

Description of Additional Supplementary Files

File Name: Supplementary Data 1
Description: Source data for main figures
