# Supplementary material for: Insights on autophagosome–lysosome tethering from structural and biochemical characterization of human autophagy factor EPG5
Source: Commun Biol. 2021 Mar 5;4:291. doi: 10.1038/s42003-021-01830-x (PMC7935953; doi:10.1038/s42003-021-01830-x)
Supplement: Supplementary file 5 — Reporting Summary [file 42003_2021_1830_MOESM5_ESM.pdf]

nature research

Corresponding author(s): Calvin K. Yip

Last updated by author(s): Jan 22, 2021

Reporting Summary

Nature Research wishes to improve the reproducibility of the work that we publish. This form provides structure for consistency and transparency in reporting. For further information on Nature Research policies, see our Editorial Policies and the Editorial Policy Checklist.

Statistics

For all statistical analyses, confirm that the following items are present in the figure legend, table legend, main text, or Methods section.

n/a	Confirmed	
☐	☒	The exact sample size (*n*) for each experimental group/condition, given as a discrete number and unit of measurement
☐	☒	A statement on whether measurements were taken from distinct samples or whether the same sample was measured repeatedly
☒	☐	The statistical test(s) used AND whether they are one- or two-sided<br>*Only common tests should be described solely by name; describe more complex techniques in the Methods section.*
☒	☐	A description of all covariates tested
☒	☐	A description of any assumptions or corrections, such as tests of normality and adjustment for multiple comparisons
☒	☐	A full description of the statistical parameters including central tendency (e.g. means) or other basic estimates (e.g. regression coefficient) AND variation (e.g. standard deviation) or associated estimates of uncertainty (e.g. confidence intervals)
☒	☐	For null hypothesis testing, the test statistic (e.g. *F*, *t*, *r*) with confidence intervals, effect sizes, degrees of freedom and *P* value noted<br>*Give P values as exact values whenever suitable.*
☒	☐	For Bayesian analysis, information on the choice of priors and Markov chain Monte Carlo settings
☒	☐	For hierarchical and complex designs, identification of the appropriate level for tests and full reporting of outcomes
☒	☐	Estimates of effect sizes (e.g. Cohen's *d*, Pearson's *r*), indicating how they were calculated

Our web collection on statistics for biologists contains articles on many of the points above.

Software and code

Policy information about availability of computer code

Data collection	All software along with the version numbers that were used for data collection in this study are stated in the Method section of the main manuscript.
Data analysis	All software that were used for data analysis in this study are stated in the Method section of the main manuscript.

For manuscripts utilizing custom algorithms or software that are central to the research but not yet described in published literature, software must be made available to editors and reviewers. We strongly encourage code deposition in a community repository (e.g. GitHub). See the Nature Research guidelines for submitting code & software for further information.

Data

Policy information about availability of data

All manuscripts must include a data availability statement. This statement should provide the following information, where applicable:

- Accession codes, unique identifiers, or web links for publicly available datasets
- A list of figures that have associated raw data
- A description of any restrictions on data availability

The 3D reconstruction of hEPG5 and atomic coordinates for GABARAPL1-hEPG5 LIR2 complex are available at the Electron Microscopy Data Bank (accession code: EMD-23120) and the Protein Data Bank (accession code: 7JHX), respectively. All source data used for generating graphs and charts in main figures are included in Supplementary Data 1. All other data are available from the corresponding author on reasonable request.

Field-specific reporting

Please select the one below that is the best fit for your research. If you are not sure, read the appropriate sections before making your selection.

☒ Life sciences ☐ Behavioural & social sciences ☐ Ecological, evolutionary & environmental sciences

For a reference copy of the document with all sections, see nature.com/documents/nr-reporting-summary-flat.pdf

Life sciences study design

All studies must disclose on these points even when the disclosure is negative.

Sample size	No sample-size calculation was performed. Sample size of n= 3 is sufficient for deriving statistics.
Data exclusions	No data were excluded from the analyses.
Replication	All experiments were performed in triplicates and the experimental findings were reproducible .
Randomization	Animal or human participants were not involved in this study, therefore randomization is not relevant to this study.
Blinding	Animal or human participants were not involved in this study, therefore blinding is not relevant to this study.

Reporting for specific materials, systems and methods

We require information from authors about some types of materials, experimental systems and methods used in many studies. Here, indicate whether each material, system or method listed is relevant to your study. If you are not sure if a list item applies to your research, read the appropriate section before selecting a response.

Materials & experimental systems

n/a	Involved in the study	
☐	☒	Antibodies
☐	☒	Eukaryotic cell lines
☒	☐	Palaeontology and archaeology
☒	☐	Animals and other organisms
☒	☐	Human research participants
☒	☐	Clinical data
☒	☐	Dual use research of concern

Methods

n/a	Involved in the study	
☒	☐	ChIP-seq
☒	☐	Flow cytometry
☒	☐	MRI-based neuroimaging

Antibodies

Antibodies used	Mouse Anti-FLAG (Sigma-Aldrich); donkey anti-mouse IRDye 680LT (LI-COR); anti-GFP (a10262; ThermoFisher); anti-mitochondrial HSP60 (128567; Abcam); chicken AlexaFluor-488 and/or mouse AlexaFluor-647 (ThermoFisher); Hoechst 33342 (ThermoFisher)
Validation	All antibodies were validated by each manufacturer listed above, except for anti-mitochondrial HSP that has been validated in the previously published paper (Nguyen et al, 2016)

Eukaryotic cell lines

Policy information about cell lines

Cell line source(s)	SF9 insect cells (Expression systems) were used for recombinant protein expressions only. Hela cell lines were used for immunofluorescence experiments; WT, LC3 TKO, GABARAP TKO and Atg8 hexa KO cells expressing mCherry-Parkin were described previously (Nguyen et al, 2016)
Authentication	None of the cell lines used were authenticated.
Mycoplasma contamination	All cell lines were tested negative for Mycoplasma.
Commonly misidentified lines (See ICLAC register)	No commonly misidentified cell lines were used in the study.
